# Supplementary material for: High-Value Plant Species Used for the Treatment of “Fever” by the Karen Hill Tribe People
Source: Antibiotics (Basel). 2020 Apr 29;9(5):220. doi: 10.3390/antibiotics9050220 (PMC7277386; doi:10.3390/antibiotics9050220)
Supplement: Supplementary file 1 [file antibiotics-09-00220-s001.pdf]

Supplementary Materials 1

High value plant species used to treat 'fever' by the Karen hill-tribe people: A meta-analysis

Methee Phumthum and Nicholas J. Sadgrove

List of data sources

Anderson, E.F. Plants and People of the Golden Triangle Ethnobotany of the Hill Tribes of Northern Thailand. Timber Press, Inc., Southwest Portland. 1993.

Junkhonkaen, J. ethnobotany of Ban Bowee, Amphoe Suan Phueng, Changwat Ratchaburi. Master thesis, Kasetsart University, Bangkok. 2012.

Junsongduang, A. Roles and importance of sacred Forest in biodiversity conservation in Mae Chaem District, Chiang Mai Province. PhD thesis, Chiang Mai University, Chiang Mai. 2014.

Kaewsangsai, S. Ethnobotany of Karen in Khun Tuen Noi Village, Mae Tuen Sub-district, Omkoi District, Chiang Mai Province. Master thesis. Chiang Mai University, Chiang Mai. 2017.

Kamwong, K. Ethnobotany of Karens at Ban Mai Sawan and Ban Huay Pu Ling, Ban Luang Sub-District, Chom Thong District, Chiang Mai Province, Master thesis. Chiang Mai University, Chiang Mai. 2010.

Kantasrila, R. Ethnobotany fo Karen at Ban Wa Do Kro, Mae Song Sub-district, Tha Song Yang District, Tak Province. Master thesis. Chiang Mai University, Chiang Mai. 2016.

Klibai, A. 2013. Self-care with indigenous medicine of long-eared Karen ethnic group: Case study Ban Mae Sin, Ban Kang Pinjai, Ban Slok, Wang Chin district, Phrae province. Master thesis, Surin Rajabhat University, Surin.

Mahawongsanan, A. Change of herbal plants utilization of the Pgn K'nyau : A case study of Ban Huay Som Poy, Mae Tia Watershed, Chom Thong District, Chiang Mai Province. Master thesis, Biology. Chiang Mai University, Chiang Mai. 2008.

Prachuabaree, L. Medicinal plants of Karang hill tribe in Baan Pong-lueg, Kaeng Krachan District, Phetchaburi Province. Master thesis, Pharmacy. Silpakorn University, Nakhonpathom. 2008.

Pongamornkul, W. An ethnobotanical study of the Karen at Ban Yang Pu Toh and Ban Yang Thung Pong, Chiang Dao district, Chiang Mai province. Chiang Mai University, Chiang Mai. 2003.

Puling, W. Ethnobotany of Karen for studying medicinal plants at Angka Noi and Mae Klang villages, Chomthong district, Chiang Mai. Bachelor degree thesis, Biology. Chiang Mai University, Chiang Mai. 2001.

Sonsupub, B. Ethnobotany of karen community in Raipa village, Huaykhayeng subdistrict, Thongphahpume district, Kanchanaburi province. Master thesis. Kasetsart University, Bangkok. 2010.

Sukkho, T. A survey of medicinal plants used by Karen people at Ban Chan and Chaem Luang Subdidtricts, Mae Chaem district, Chiang Mai province. Chiang Mai University, Chiang Mai. 2008.

Sutjaritjai, N.; Wangpakapattanawong, P.; Balslev, H.; Inta, A. Sutjaritjai, N., P. Wangpakapattanawong, H. Balslev and A. Inta (2019). "Traditional Uses of Leguminosae among the Karen in Thailand. Plants. 2019; 8(12). https://doi.org/10.3390/plants8120600
Trisonthi, S.; Trisonthi, P. Ethnobotany of Karen in Mae Hae Nua village, Mae Na Jorn subdistrict, Mae Chaem district, Chiang Mai. Scientific report. CU Library, Bangkok. 1995.

Winijchaiyanan, P. Ethnobotany of Karen in Chiang Mai. Master thesis, Biology. Chiang Mai University Library, Chiang Mai. 1995.

Supplementary Materials 2

High value plant species used to treat 'fever' by the Karen hill-tribe people: A meta-analysis

Methee Phumthum and Nicholas J. Sadgrove

Table S1. CI values of medicinal plant families used for fever treatment by 25 Karen villages in Thailand.

Family	CI
Acanthaceae	0.4
Acoraceae	0.12
Adoxaceae	0.04
Amaryllidaceae	0.08
Anacardiaceae	0.04
Annonaceae	0.08
Apiaceae	0.08
Apocynaceae	0.24
Araceae	0.12
Asparagaceae	0.16
Asteraceae	0.44
Bignoniaceae	0.12
Burseraceae	0.04
Cannabaceae	0.08
Chloranthaceae	0.08
Clusiaceae	0.04
Euphorbiaceae	0.24
Hernandiaceae	0.04
Hypoxidaceae	0.04
Iridaceae	0.08
Lamiaceae	0.32
Lauraceae	0.16
Leguminosae	0.72
Loranthaceae	0.12
Malvaceae	0.2

Melastomataceae	0.12
Meliaceae	0.12
Menispermaceae	0.16
Moraceae	0.04
Musaceae	0.04
Oleaceae	0.04
Phyllanthaceae	0.12
Plantaginaceae	0.16
Plumbaginaceae	0.04
Poaceae	0.08
Polygalaceae	0.04
Polypodiaceae	0.04
Ranunculaceae	0.04
Rubiaceae	0.16
Rutaceae	0.36
Salicaceae	0.04
Santalaceae	0.04
Saururaceae	0.04
Simaroubaceae	0.12
Smilacaceae	0.08
Solanaceae	0.04
Staphyleaceae	0.04
Tectariaceae	0.04
Theaceae	0.08
Thymelaeaceae	0.04
Vitaceae	0.04
Xanthorrhoeaceae	0.04

Table S2 Plant part used and CI values of medicinal plant families used for fever treatment by 25 Karen villages in Thailand

Species	Part used	CI
Acacia rugata (Lam.) Fawc. & Rendle	Leaves	0.08
Acalypha spiciflora Burm.f.	Stems	0.04
Acorus calamus L.	Entire plant	0.12
Aglaia lawii (Wight) C.J.Saldanha	Roots	0.04
Allium ascalonicum L.	Stems	0.04
Aloe vera (L.) Burm.f.	Leaves	0.04
Alstonia rostrata C. E. C. Fisch.	Barks	0.08
Alstonia scholaris (L.) R. Br.	Barks	0.08
Andrographis paniculata (Burm.f.) Nees	Entire plant	0.08
Aquilaria crassna Pierre ex Lecomte	Barks	0.04
Archidendron clypearia (Jack) I.C.Nielsen	Leaves	0.08
Artemisia atrovirens Hand.-Mazz.	Entire plant	0.04
Aspidistra elatior Blume		0.04
Azadirachta indica A.Juss.	Leaves	0.04
Blumea balsamifera (L.) DC.	Entire plant	0.08

Caesalpinia sappan L.		0.04
Calophyllum polyanthum Wall. ex Planch. & Triana	Barks	0.04
Camellia sinensis (L.) Kuntze	Leaves	0.04
Cassytha filiformis L.	Entire plant	0.08
Cayratia pedata (Lam.) Gagnep.	Entire plant	0.04
Celtis tetrandra Roxb.	Stems	0.04
Celtis timorensis Span.	Barks	0.04
Litsea monopetala (Roxb.) Pers.	Shoot	0.04
Mallotus philippensis (Lam.) Müll.Arg.	Stems	0.04
Mayodendron igneum (Kurz) Kurz	Barks	0.04
Melastoma sanguineum Sims.	Stems	0.04
Melicope glomerata (W. G. Craib) T.G. Hartley	Stems	0.12
Memecylon pauciflorum Blume	Leaves	0.04
Micromelum integerrimum (Buch.-Ham. ex DC.) Wight & Arn. ex M. Roem.	Leaves	0.04
Millingtonia hortensis L.f.	Aerial parts	0.04
Mimosa diplotricha Sauvalle	Entire plant	0.04
Mimosa pigra L.	Entire plant	0.08
Mimosa pudica L.	Entire plant	0.16
Molineria capitulata (Lour.) Herb.	Roots	0.04
Musa acuminata Colla	Stems	0.04
Nyctocalos brunfelsiiflora Teijsm. & Binn.	Entire plant	0.04
Oenanthe javanica (Blume) DC.	Shoot	0.04
Osbeckia chinensis L.	Entire plant	0.04
Peliosanthes caesia J.M.H.Shaw	Entire plant	0.04
Peliosanthes macrophylla Wall. ex Baker	Roots	0.04
Phlogacanthus curviflorus (Wall.) Nees	Entire plant	0.04
Phyllanthus amarus Schumach. & Thonn.	Entire plant	0.12
Physalis minima L.	Entire plant	0.04
Picrasma javanica Blume	Barks	0.08
Plantago major L.	Leaves	0.04
Platycerium wallichii Hook.	Entire plant	0.04
Plectranthus amboinicus (Lour.) Spreng.	Leaves	0.04
Plumbago indica L.	Entire plant	0.04
Plumeria obtusa L.	Leaves	0.04
Polygala arillata Buch.-Ham. ex D. Don	Roots	0.04
Pothos chinensis (Raf.) Merr.	Entire plant	0.08
Pothos scandens L.	Entire plant	0.04

Psychotria yunnanensis Hutch.	Entire plant	0.04
*Pteridrys syr*matica (Willd.) C. Chr. & Ching	Rhizome	0.04
Rotheca serrata (L.) Steane & Mabb.	Entire plant	0.08
Sambucus javanica Blume		0.04
Sarcandra glabra (Thunb.) Nakai	Entire plant	0.04
Scadoxus multiflorus (Martyn) Raf.	Stems	0.04
Schima wallichii Choisy	Roots	0.04
Scoparia dulcis L.	Roots	0.12
Scurrula ferruginea (Jack) Danser	Entire plant	0.04
Senna hirsuta (L.) H.S.Irwin & Barneby	Entire plant	0.04
Sida acuta Burm.f.	Entire plant	0.08
Sida cordifolia L.	Entire plant	0.04
Sida rhombifolia L.	Entire plant	0.08
Smilax glabra Roxb.	Roots	0.04
Smilax luzonensis C.Presl	Rhizome	0.04
Spondias pinnata (L. f.) Kurz	Barks	0.04
Streblus asper Lour.	Leaves	0.04
Strobilanthes cusia (Nees) Kuntze	Leaves	0.08
Tadehagi triquetrum (L.) H.Ohashi	Entire plant	0.08
Tamarindus indica L.	Shoot	0.04
Thunbergia coccinea Wall.	Entire plant	0.04
Thunbergia laurifolia Lindl.	Aerial parts	0.08
Thysanolaena latifolia (Roxb. ex Hornem.) Honda	Roots	0.04
Tiliacora triandra Diels	Roots	0.04
Tinospora baenzigeri Forman	Stems	0.04
Tinospora crispa (L.) Hook. f. & Thomson	Stems	0.04
Triadica cochinchinensis Lour.	Stems	0.04
*Turpinia pom*ifera (Roxb.) DC.	Roots	0.04
Viscum articulatum Burm. f.	Entire plant	0.04
Vitex trifolia L.	Leaves	0.08
